# Astrocytes modulate thalamic sensory processing via mGlu2 receptor activation

**DOI:** 10.1016/j.neuropharm.2017.04.019

**Published:** 2017-07-15

**Authors:** C.S. Copeland, T.M. Wall, R.E. Sims, S.A. Neale, E. Nisenbaum, H.R. Parri, T.E. Salt

**Affiliations:** a Institute of Ophthalmology, University College London, 11-43 Bath Street, London, EC1V 9EL, UK; b St George's, University of London, Cranmer Terrace, London, SW17 0RE, UK; c Eli Lilly and Company, 893 S Delaware Street, Indianapolis, IN 46285, USA; d School of Life and Health Sciences, Aston University, Birmingham, B4 7ET, UK; e Neurexpert Limited, Kemp House, 152-160 City Road, London, EC1V 2NX, UK

**Keywords:** Astrocyte, Metabotropic glutamate receptor subtype 2, Synaptic inhibition, Thalamus, Thalamic reticular nucleus, AMPA, α-Amino-3-hydroxy-5-methyl-4-isoxazolepropionic acid, CNQX, 6-cyano-7-nitroquinoxaline-2,3-dione, DL-APV, DL-2-Amino-5-phosphonopentanoic acid, DMSO, dimethyl sulfoxide, GABA, gamma amino butyric acid, i.p., intraperitoneal, LY341495, (2*S*)-2-Amino-2-[(1*S*,2*S*)-2-carboxycycloprop-1-yl]-3-(xanth-9-yl)propanoic acid, LY354740, (1S,2S,5R,6S)-2-Aminobicyclo[3.1.0]hexane-2,6-dicarboxylic acid, LY487379, 2,2,2-Trifluoro-N-[4-(2-methoxyphenoxy)phenyl]-N-(3-pyridinylmethyl)ethanesulfonamide hydrochloride, mGlu, metabotropic glutamate, mGlu2, metabotropic glutamate receptor subtype 2, mGlu3, metabotropic glutamate receptor subtype 3, mIPSC, miniature inhibitory post-synaptic current, NaCl, sodium chloride, NIH, National Institutes of Health, NMDA, N-methly-d-aspartate, PAM, positive allosteric modulator, PSTH, post-stimulus time histogram, ROI, region of interest, SEM, standard error of the mean, SR101, Sulforhodamine 101, TRN, thalamic reticular nucleus, TTX, tetrodotoxin, VB, ventrobasal thalamus

## Abstract

Astrocytes possess many of the same signalling molecules as neurons. However, the role of astrocytes in information processing, if any, is unknown. Using electrophysiological and imaging methods, we report the first evidence that astrocytes modulate neuronal sensory inhibition in the rodent thalamus.

We found that mGlu2 receptor activity reduces inhibitory transmission from the thalamic reticular nucleus to the somatosensory ventrobasal thalamus (VB): mIPSC frequencies in VB slices were reduced by the Group II mGlu receptor agonist LY354740, an effect potentiated by mGlu2 positive allosteric modulator (PAM) LY487379 co-application (30 nM LY354740: 10.0 ± 1.6% reduction; 30 nM LY354740 & 30 μM LY487379: 34.6 ± 5.2% reduction).

We then showed activation of mGlu2 receptors on astrocytes: astrocytic intracellular calcium levels were elevated by the Group II agonist, which were further potentiated upon mGlu2 PAM co-application (300 nM LY354740: ratio amplitude 0.016 ± 0.002; 300 nM LY354740 & 30 μM LY487379: ratio amplitude 0.035 ± 0.003).

We then demonstrated mGlu2-dependent astrocytic disinhibition of VB neurons *in vivo*: VB neuronal responses to vibrissae stimulation trains were disinhibited by the Group II agonist and the mGlu2 PAM (LY354740: 156 ± 12% of control; LY487379: 144 ± 10% of control). Presence of the glial inhibitor fluorocitrate abolished the mGlu2 PAM effect (91 ± 5% of control), suggesting the mGlu2 component to the Group II effect can be attributed to activation of mGlu2 receptors localised on astrocytic processes within the VB.

Gating of thalamocortical function via astrocyte activation represents a novel sensory processing mechanism. As this thalamocortical circuitry is important in discriminative processes, this demonstrates the importance of astrocytes in synaptic processes underlying attention and cognition.

## Highlights

• Thalamic inhibition is mediated by both neuronal and astrocytic mechanisms.
• Group II mGlu receptor (mGlu2/3) activation can modulate this thalamic inhibition.
• Thalamic astrocytes can be activated upon mGlu2 receptor stimulation.
• This process may enable relevant activity to be discerned from background noise.
• Targeting astrocytic mGlu2 receptors may therefore affect attention and cognition.

## 1 Introduction

The thalamic reticular nucleus (TRN) is responsible for ensuring synchronous activity across specific thalamo-cortical circuits required for sensory perception or the preparation and execution of distinct motor and/or cognitive tasks. It is therefore imperative to ascertain how inhibition from the TRN to thalamic nuclei is controlled to understand how neurophysiological disease states associated with TRN malfunction precipitate (Huguenard, 1999, Rub et al., 2003, Barbas and Zikopoulos, 2007, Pinault, 2011).

**Fig. 1:**
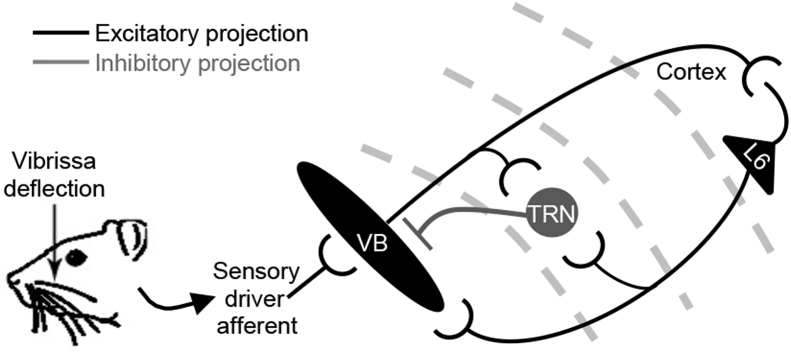
**Thalamic circuitry underlying responses to vibrissal deflection.** Branching collaterals from excitatory thalamocortical and corticothalamic axons (black), which originate from functionally linked topographical areas in the thalamus/cortex, innervate the TRN (Ohara and Lieberman, 1985, Shosaku et al., 1989, Rouiller et al., 1998, Kakei et al., 2001), and the TRN sends a reciprocal inhibitory projection (grey) back to the thalamic area from which it receives its thalamocortical innervation (Jones, 1985, Salt, 1989, Shosaku et al., 1989, Pinault and Deschenes, 1998, Salt and Turner, 1998, Crabtree, 1999).

The TRN surrounds the entire anteroposterior extent of the dorsal thalamus, meaning all thalamo-cortical and cortico-thalamic projections must pass through and make connections with its mesh of inhibitory interneurons (Houser et al., 1980, Jones, 1985) (Fig. 1). This strategic localisation between thalamus and cortex enables the TRN to mediate coherent activity patterns within the thalamo-cortico-thalamic excitatory loop by providing both feedback and feedforward inhibition to thalamic nuclei upon thalamo-cortical and cortico-thalamic input, respectively (Shosaku et al., 1989) (Fig. 1). The Group II metabotropic glutamate (mGlu) receptors (mGlu2/3) modulate physiologically-evoked responses in the somatosensory ventrobasal thalamic nucleus (VB) by reducing inhibition from the TRN (Salt and Turner, 1998, Copeland et al., 2012), with the mGlu2 component to this Group II effect likely activated by glutamate spillover upon physiological sensory stimulation (Copeland et al., 2012).

VB astrocytes *in vitro* can respond to sensory afferent stimulation with an elevation in intracellular calcium (Parri et al., 2010), in accordance with astrocytic activation in other brain regions (Porter and McCarthy, 1996, Grosche et al., 1999, D'Ascenzo et al., 2007). These elevations can initiate release of gliotransmitters including glutamate (Fellin et al., 2004), d-serine (Panatier et al., 2006), adenosine triphosphate (Guthrie et al., 1999) and adenosine (Winder et al., 1996), with subsequent modulation of neuronal excitability and synaptic transmission (Fellin et al., 2004, Serrano et al., 2006). Astrocytic processes co-localise with sensory and TRN afferent terminals around the soma and proximal dendrites of VB neurons (Ralston, 1983, Ohara and Lieberman, 1993); thus, it is important to understand how astrocytes are activated as concomitant gliotransmission may represent a significant mechanism in the regulation of thalamo-cortical network function via modulation of the TRN-VB synapse.

Here, by firstly using *in vitro* electrophysiology we confirmed the presence of an mGlu2 component to the overall Group II mGlu receptor effect on inhibitory synaptic transmission from the TRN to the VB, as previously indicated *in vivo* (Copeland et al., 2012). By then using *in vitro* calcium imaging, which enabled the identification of the cellular foundation supporting this mechanism, the mGlu2 component was identified as astrocyte-dependent: mGlu2 receptor activation elicited elevations in astrocytic (but ***not*** neuronal) intracellular calcium - a novel mechanism of astrocyte activation. Finally, we identified VB neurons responsive to trains of single vibrissa stimuli *in vivo* and applied selective compounds locally. We delineate that the mGlu2 receptor astrocyte-dependent mechanism contributes to the modulation of sensory transmission in a physiological context. Together, the data indicate that the mGlu2 component to the Group II mGlu receptor effect is purely astrocyte-dependent, making astrocytes an integral signalling intermediary in sensory processing.

## 2 Material and methods

### 2.1 Ethical approval

All experimental conditions and procedures were either in accordance with the National Institutes of Health (NIH) regulations of animal care covered in the Principles of Laboratory Animal Care, NIH publication 85–23, revised 1985, and were approved by the Eli Lilly and Company Institutional Animal Care and Use Committee, or were approved by the Home Office (UK) and were in accordance with the UK Animals (Scientific Procedures) Act 1986 and associated guidelines.

### 2.2 *I**n vitro* electrophysiology

#### 2.2.1 Animals

Male Sprague-Dawley rats (12–18 days old; Harlan, Indianapolis, USA, n = 10) were deeply anaesthetised with 4.0% isoflurane and decapitated into a container of crushed ice.

#### 2.2.2 Slice preparation and maintaining solutions

The brain was quickly removed and placed in an oxygenated, ice cold beaker of slicing solution which contained (in mM): 110 NaCl; 10 MgCl_2_; 2 KCl; 26 NaHCO_3_; 1.25 NaH_2_PO_4_; 0.5 CaCl_2_; 10 HEPES and 15 glucose (pH adjusted to 7.45 with NaOH, osmolarity was 308–312 mOsm). After cooling in slicing solution for 2–3 min, the whole brain was blocked (portions of anterior and posterior tissue removed) using a razor blade and then glued to the microslicer (DTK Zero 1, DSK) tray using cyanoacrylate. The tray containing the blocked and mounted brain was filled with oxygenated, ice cold slicing solution and serial, coronal sections were cut at a thickness of 300 μm. Slices were then placed in a larger recovery chamber containing oxygenated slicing solution at room temperature (18–20 °C). The recovery chamber was in a large water bath, which was initially at room temperature. After a 10 min period, 500 μL of 0.5 M CaCl_2_ solution was slowly added to the recovery chamber (500 ml volume) to increase the calcium concentration to 1 mM. The water bath was then turned on and the temperature was monitored inside the recovery chamber. The recovery chamber temperature was allowed to reach 33–34 °C for a period of approximately 30 min, after which the water bath was turned off and the recovery chamber was allowed to slowly return to room temperature (18–20 °C). Slices were used for recording after at least 1 h of recovery time.

#### 2.2.3 Recording conditions

Slices were placed in a superfusion chamber mounted on a Nikon Eclipse FN-1 microscope. Neurons within the VB area of the thalamus were visualized using IR/DIC water immersion optics. The recording solution was composed of (in mM): 115 NaCl; 1.5 MgCl_2_; 5 KCl; 26 NaHCO_3_; 1.25 NaH_2_PO_4_; 10 HEPES; 2 CaCl_2_ and 15 glucose at pH 7.45, oxygenated with carbogen gas (95%O_2_/5%CO_2_) and osmolarity of 300–305 mOsm. The brain slice in the chamber was continually superfused at a rate of 3 mL/min with oxygenated recording solution (18–20 °C). Compound containing solutions were applied to the slice via whole chamber superfusion. Glass recording electrodes were filled with (in mM): 140 CsCl; 1 MgCl_2_; 10 HEPES; 3 NaATP; 0.3 NaGTP; 1 Cs-EGTA at pH 7.2 and osmolarity adjusted to 294–300 mOsm and had a resistance of 2–4 MΩ.

#### 2.2.4 Experimental protocol

Visualized neurons were patch clamped in whole cell configuration (Multiclamp 700B, MDS) and access resistance (Ra) was evaluated in voltage clamp mode. A gapfree protocol (Clampex V10, MDS) with a holding potential of −70 mV was used to record miniature synaptic events until the access resistance and holding current were stable in recording solution only. The slice was then superfused with recording solution containing 10 μM 6-cyano-7-nitroquinoxaline-2,3-dione (CNQX; Tocris), 50 μM DL-2-Amino-5-phosphonopentanoic acid (DL-APV; Tocris) and 0.5 μM tetrodotoxin (TTX; Abcam) to block the AMPA- and NMDA-evoked miniature and large amplitude events due to direct action potential firing of inhibitory neurons, respectively, leaving only the GABA mediated miniature synaptic events (confirmed in preliminary experiments by complete blockade of remaining synaptic events with 10 μM bicuculline). LY354740, LY341495 and LY487379 (all made in-house) stocks were made in 100% DMSO at 1000× the desired working concentration. Compounds were diluted into the recording solution containing CNQX, APV and TTX immediately before application to the brain slice. All solutions applied to the brain slices contained 0.1%–0.2% DMSO. DMSO content was matched between solutions for each experimental protocol. Compound treatment periods were from 10 to 12 min in duration.

#### 2.2.5 Data collection and statistical analysis

The frequency of the GABAergic miniature synaptic events was determined during the final 5 min of each treatment period (baseline, 30 nM LY354740, 100 nM LY354740, 100 nM LY354740 + 100 nM LY341495, 30 nM LY354740 + 30 μM LY487379, and 30 μM LY478379) using the MiniAnalysis program (V6.0.4, Synaptosoft). Inter-event intervals were calculated and plotted as cumulative fraction histograms for each treatment group. The Kolmogorov-Smirnov test was performed on the inter-event interval cumulative fractions to determine statistical significance of compound effects on spontaneous GABAergic synaptic event activity.

### 2.3 Intracellular calcium imaging

#### 2.3.1 Animals

Male juvenile Wistar rats and mice (10–16 days old; n = 5; bred in house) were killed by halothane overdose followed by cervical dislocation. IP3 R2 Knockout Mice (Ju Chen, UCSD) were bred from founder mice kindly obtained from A. Araque, Instituto Cajal, Madrid. Mice were bred on a C57Bl6 background. WTs (-/-) were bred from Heterozygous (±) bred pairs. Genotyping was conducted by Transnetyx (Cordova, TN, USA).

#### 2.3.2 Slice preparation and maintaining solutions

Slices were prepared as described previously (Parri and Crunelli, 2001). Briefly, following removal from the skull, the brain was glued with cyanoacrylate adhesive to a metal block and submerged in the bath of Microm MV (Zeiss, Welwyn Garden City, UK) tissue slicer. The bathing solution was of the following composition (in mM): NaCl 120, NaHCO_3_ 16, KCl 1, KH_2_PO_4_ 1.25, MgSO_4_ 5, CaCl_2_ 1, glucose 10, and was maintained at 5 °C. Thalamic slices (350 μm) were cut in the horizontal plane, and then stored in a 95% O_2_/5% CO_2_ bubbled solution of identical composition at room temperature.

Following a 1 h recovery period, experiments were performed in a solution of the following composition (in mM): NaCl 120, NaHCO_3_ 16 or 25, KCl 2, KH_2_PO_4_ 1.25, MgSO_4_ 1, CaCl_2_ 2, glucose 10, at room temperature (20–24 °C), unless otherwise stated. TTX (0.5 μm) was included in the perfusate to block sodium currents in VB neurons (Parri and Crunelli, 1998). Compounds LY354740, LY487379 and MPEP were obtained from Tocris (Bristol, UK), Suramin from Sigma-Aldrich.

#### 2.3.3 Fluorescence imaging

Slices were loaded with either Fura-2 or Fluo4-AM (Invitrogen) dye (5 μM with 0.01% pluronic acid) after a post-cutting recovery period of 1 h. Fluo4 was routinely used in experiments to monitor Group II mGlu receptor activation, Fura-2 was used in dose response determination experiments where comparison of repetitive drug applications in the same astrocytes was required. Astrocytes and neurons were distinguishable by their morphological profiles: VB neurons have large somas (18 μm diameter), with 3–4 dendrites; astrocytes have much smaller somas (∼8 μm) with nebulous processes (Parri et al., 2001). Slices were also loaded with 1 μM Sulforhodamine 101 (SR101), according to published *in vitro* methods (Kafitz et al., 2008) for verification of astrocyte identity. The recording chamber and manipulators were mounted on a motorized moveable bridge (Luigs and Neumann) and fluorescence dyes were excited using an Optoscan monochromator system, fitted to a Nikon FN1 upright microscope; filter cubes for selective Fura-2, Fluo4 and SR101 imaging were obtained from Chroma. Images of slice areas of 444  μm × 341  μm were routinely acquired every 5s with a ×20 objective lens (NA = 0.8) using an ORCA ER CCD camera (Hamamatsu) and analysed using Simple PCI software (Hamamatsu). Fluorescence values over time for specific regions of interest (ROIs) were exported and analysed using Sigmaplot (Systat). The number of events during a recording was determined by identifying events where amplitude exceeded 2 standard deviations of baseline variations. For determination of amplitude changes, the absolute ratio or ΔF increases in the different conditions in the same cells were directly compared, so providing an internal control.

#### 2.3.4 Statistical analysis

All quantitative data are expressed in the text as mean (±SEM). Statistical tests included Student's t-test and the Kolmogorov–Smirnov test for cumulative population distributions, as indicated.

### 2.4 In vivo single neuron recording and iontophoresis

#### 2.4.1 Animals

All experiments were conducted using adult male Wistar rats (340–540 g, n = 18). Animals (Harlan, UK) were housed on a 12 h light/dark cycle with food and water *ad libitum*.

#### 2.4.2 Surgery

Animals were anaesthetised with urethane (1.2  g/kg intraperitoneal [i.p.] injection) and were prepared for recording as previously described (Salt, 1987, Salt, 1989). Throughout the experiments, electroencephalogram and electrocardiogram were monitored. Additional urethane anaesthetic was administered i. p. as required, and the experiment was terminated with an overdose of the same anaesthetic.

#### 2.4.3 Recording and iontophoresis

Seven-barrel recording and iontophoretic glass pipettes were advanced into the VB. Extracellular recordings were made from single VB neurons responsive to somatosensory input through the central barrel (filled with 4 M sodium chloride [NaCl]). Iontophoretic drug applications were performed using the outer barrels (Salt, 1987, Salt, 1989). On each occasion, one of the outer barrels was filled with 1 M NaCl for current balancing. The remaining outer barrels each contained one of the following substances: N-methyl-d-aspartate (NMDA; 50 mM, pH8.0 in 150 mM NaCl), (1S,2S,5R,6S)-2-Aminobicyclo[3.1.0]hexane-2,6-dicarboxylic acid (LY354740; 5 mM, pH8.0 in 75 mM NaCl), dl-Fluorocitric Acid (10 mM, pH8.0 in 75 nM NaCl) as Na^+^ salts, ejected as anions, with 2,2,2-Trifluoro-N-[4-(2-methoxyphenoxy)phenyl]-N-(3-pyridinylmethyl)ethanesulfonamide hydrochloride (LY487379; 1 mM, pH6.0, in 1% dimethyl sulfoxide [DMSO], 75 mM NaCl ejected as cations. All compounds were prevented from diffusing out of the pipette by using a retaining current (10–20 nA) of opposite polarity to that of the ejection current. Compounds were ejected within a current range ensured to produce a sub-maximal effect on sensory inhibition (LY354740 6nA-50nA; LY487379 50nA-100nA) or neuronal excitation (NMDA 35nA-85nA). Fluorocitrate was obtained from Sigma (St Louis, MO, USA), with all other compounds obtained from Tocris (Bristol, UK). It is of importance to note that both the Group II orthosteric agonist LY354740 (Monn et al., 1997) and the mGlu2 positive allosteric modulator (PAM) LY487379 (Schaffhauser et al., 2003) used in this study possess a higher selectivity for their receptor targets than the prototypical Group II orthosteric antagonist (2*S*)-2-Amino-2-[(1*S*,2*S*)-2-carboxycycloprop-1-yl]-3-(xanth-9-yl)propanoic acid (LY341495) (Kingston et al., 1998), which has been demonstrated to have antagonistic properties at both the Group II and Group III mGlu receptors in a similar iontophoretic *in vivo* study (Cirone and Salt, 2001).

#### 2.4.4 Stimulation protocols

Neurons were identified as VB neurons on the basis of stereotaxic location (Paxinos and Watson, 1998) and responses to vibrissa deflection. Vibrissa deflection was performed using fine air-jets directed through 23 gauge needles mounted on micro-manipulators positioned and orientated close to the vibrissa to ensure deflection of a single vibrissa was achieved. Air-jets were electronically gated with solenoid valves that produced a rising air pulse at the vibrissa 8 ms after switching. Response latencies were calculated from the start of the gating pulse. Using such an approach it is possible to use air-jets to evoke an excitatory response from stimulation of a single vibrissa, as described previously (Salt, 1989). Prior to the beginning of each of the experimental protocols described below, the ‘principal’ vibrissa (i.e. the vibrissa at the centre of the receptive field) for each neuron was identified. All neurons recorded from were quiescent.

##### 2.4.4.1 Protocol 1

The effects of the selective glial inhibitor fluorocitrate on VB neuronal responses to train stimulation of vibrissae and iontophoretic NMDA application.

Cycles of sensory and NMDA stimulation were established and repeated continuously whilst recording from neurons. Cycles (60s long) contained two types of stimuli consisting of 500–1000ms duration trains (5–10 Hz) of air-jets directed at the principal vibrissa, repeated 4 times with a 4s interstimulus interval, followed by a single iontophoretic NMDA application (10s), which was timed to provide a 15s interval either side of the sensory stimulations. After several control cycles displaying consistent VB neuronal responses had been recorded, fluorocitrate was iontophoretically ejected for 5–12min as required until a consistent effect of fluorocitrate was observed. An inter-stimulus interval of 4s is sufficient to ensure that any post-stimulus effects from either stimulus type are no longer apparent upon subsequent stimulation (Salt, 1989, Turner and Salt, 2003).

##### 2.4.4.2 Protocol 2

The effects of the selective glial inhibitor fluorocitrate on Group II mGlu receptor modulation of sensory inhibition.

Cycles of sensory stimulation (10s long) were established and repeated continuously whilst recording from neurons. Cycles contained one type of stimulus consisting of 500–1000ms duration trains (5–10 Hz) of air-jets directed at the principal vibrissa. After several control cycles displaying consistent neuronal responses had been recorded, LY487379 and LY354740 were iontophoretically ejected for 2–15 min s as required, under normal conditions and in the presence of fluorocitrate. After cessation of compound ejection, sensory stimulation cycles were continued until VB neuronal responses had returned to their respective control levels.

#### 2.4.5 Data collection and statistical analysis

Throughout the study, extracellular single neuron action potentials were gated, timed and counted using a window discriminator, a CED1401 interface and Spike2 software (Cambridge Electronic Design, Cambridge, UK), which recorded the output from the iontophoresis unit and also triggered the iontophoretic and sensory stimuli sequences. Data were analysed by plotting post-stimulus time histograms (PSTHs) from these recordings by counting the spikes evoked by either NMDA ejection or sensory stimulation. Data are expressed as a percentage of control responses prior to compound application (±SEM). Comparisons were made using Wilcoxon matched-pairs test (*p* < 0.05).

## 3 Results

The pharmacological compounds used in this study are clearly crucial to the interpretation of the results. LY354740 is the best-studied selective Group II orthosteric agonist (Monn et al., 1997, Schoepp et al., 2003), and has been used extensively to reveal Group II mGlu receptor function in both behavioural (Schoepp et al., 2003; Nordquist et al., 2008) and *in vitro/vivo* physiological (Flor et al., 2002; Moldrich et al., 2003; Copeland et al., 2012) assays in rodent and human CNS models. LY487379 is a highly selective mGlu2 PAM, which possesses no intrinsic agonist activity but does enhance responses to submaximal glutamate without activity at other receptors or ion channels (Johnson et al., 2003). LY487379 has been used in a number of pharmacological assays, including behavioural and *in vitro/vivo* electrophysiological studies in the rodent CNS (Schaffhauser et al., 2003; Galici et al., 2005; Poisik et al., 2005; Harich et al., 2007; Hermes and Renaud, 2011; Nikiforuk et al., 2010; Copeland et al., 2012). The orthosteric antagonist LY341495 has a relatively high selectivity with a nanomolar potency for Group II mGlu receptor, with submicromolar potencies at all other mGlu receptor subtypes (Kingston et al., 1998; Schoepp et al., 1999). However, the parameters used for LY341495 in this study have been demonstrated previously to produce selective antagonism for the Group II mGlu receptors (Kingston et al., 1998).

### 3.1 mGlu2 receptors modulate synaptic transmission at the TRN-VB synapse

**Fig. 2:**
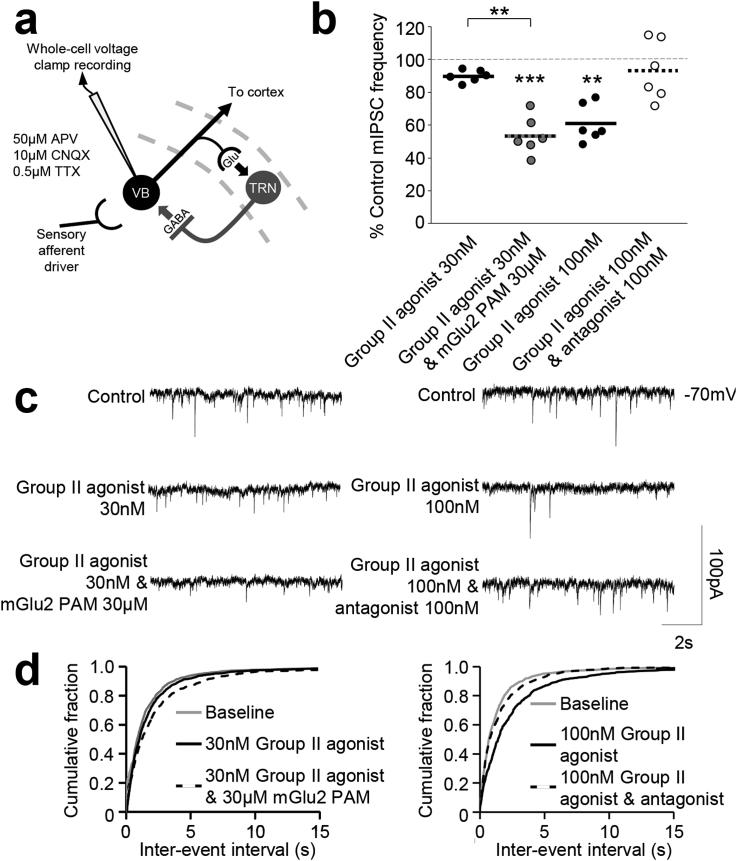
**The Group II mGlu receptor effect on spontaneous presynaptic quantal release events includes an mGlu2 receptor-mediated component. a** Circuitry between the TRN and VB with recording site indicated. **b** Effects of the Group II agonist LY354740 (30 nM) alone or in conjunction with the mGlu2 PAM LY487379 (30ìM) on the total number of spontaneous mIPSC events (final 5 min bin) in the VB. Specificity of the Group II agonist effect was confirmed upon its reversal using the Group II antagonist LY341495 (100 nM). **c** Traces from individual neurons illustrating the mean responses of neurons to the same conditions as described in **b**. **d** Effects of the same compound application combinations on the cumulative fraction of the calculated inter-event intervals of the spontaneous mIPSCs in the VB. **p < 0.001; ***p < 0.0001; Glu – glutamate.

Group II mGlu receptor activation has been previously demonstrated to depress VB neuron inhibitory postsynaptic potentials (IPSPs) evoked upon stimulation of the TRN (Turner and Salt, 2003), and an mGlu2 component to this Group II effect was recently described in an *in vivo* study (Copeland et al., 2012). Therefore, we first determined whether mGlu2 receptor activation is able to modulate inhibitory synaptic transmission at the TRN-VB synapse. One component that would contribute to IPSP depression is direct inhibition of GABAergic vesicle fusion with the presynaptic TRN membrane. By recording miniature inhibitory postsynaptic currents (mIPSCs) it is possible to examine the frequency of spontaneous presynaptic quantal release events and so detect changes in transmitter release in the absence of evoked synaptic activity. In the absence of endogenous mGlu2 receptor activation, a sub-maximal concentration (30 nM) of the Group II agonist LY354740 was able to reduce mIPSC frequency compared to baseline when applied alone (10.0 ± 1.6% reduction compared to control, n = 6 from 6 slices, Fig. 2). Application of the mGlu2 PAM LY487379 alone had no effect on mIPSC frequency (data not shown). By nature of design, PAMs potentiate the action of orthosteric agonists, without themselves possessing any intrinsic agonist activity (Johnson et al., 2003). This lack of effect of the PAM in this preparation is therefore unsurprising as there is likely no baseline activation of mGlu2 receptors under these conditions. However, when the mGlu2 PAM was co-applied with the sub-maximal concentration of Group II agonist, a significant additional reduction in mIPSC frequency was observed (30 nM LY354740 & 30 μM LY487379: 34.6 ± 5.2% reduction, n = 6 from 6 slices, *p* < 0.001, Fig. 2), comparable to that seen upon maximal agonist effect (100 nM LY354740: 39.1 ± 4.7% reduction compared to control, n = 6 from 6 slices, *p* < 0.001; Fig. 2). The Group II mGlu receptor effect on mIPSC frequency was confirmed by its reversal upon Group II orthosteric antagonist LY341495 co-application (100 nM LY354740 & 100 nM LY341495: 6.6 ± 7.5% reduction in mIPSC frequency compared to control, n = 6 from 6 slices, *p* < 0.01, Fig. 2). Taken together these data indicate that there is indeed an mGlu2 component to the Group II mGlu receptor effect on GABAergic transmission at the TRN-VB synapse. Ultrastructural studies indicate that TRN terminals exclusively express the mGlu3 receptor subtype (Tamaru et al., 2001), while VB astrocytes express both mGlu2 and mGlu3 (Ralston, 1983, Ohara and Lieberman, 1993, Liu et al., 1998, Mineff and Valtschanoff, 1999). We therefore sought to confirm functional expression of astrocytic mGlu2 receptors.

### 3.2 mGlu2 receptors activate astrocytes in the VB

**Fig. 3:**
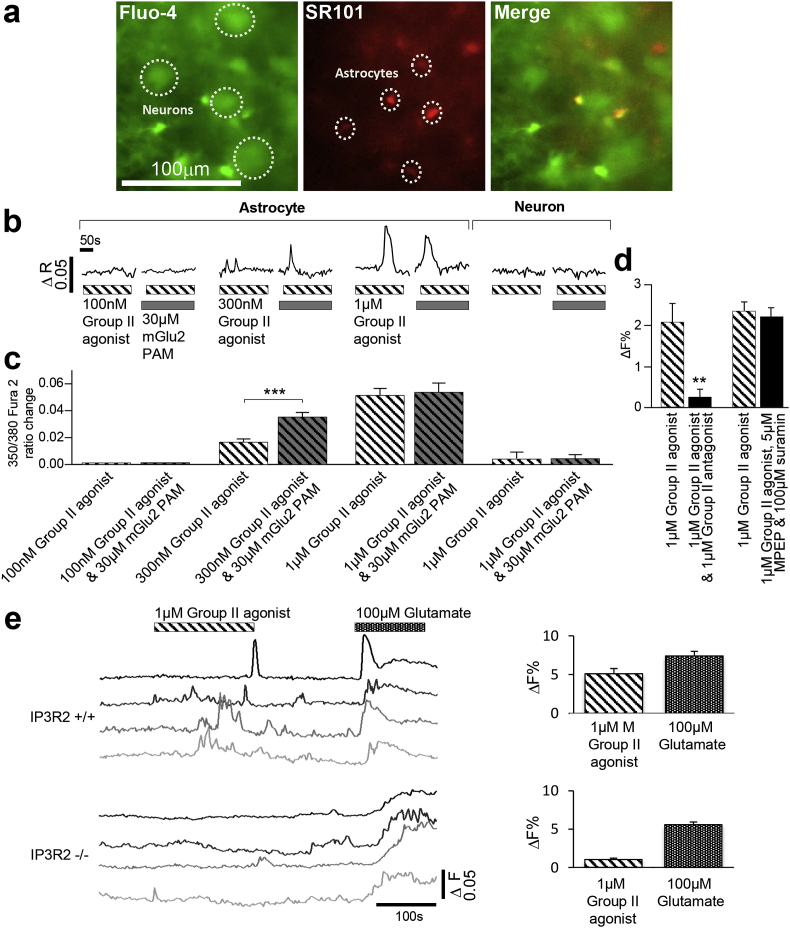
**mGlu2 receptor activation can elicit increases in astrocytic intracellular calcium levels. a** Images from a slice loaded with Fluo-4-AM for calcium imaging, and SR101 for astrocyte differentiation. Identified astrocytes and neurons are indicated. **b** Traces displaying transient intracellular calcium elevations in an astrocyte in response to application of increasing concentrations of the Group II agonist LY354740 either alone or in conjunction with the mGlu2 PAM LY487379. Two traces on the right display ratio over time for example a neuron. c Bargraphs summarise results from a number of experiments corresponding to the illustrative traces above in **b**. (Astrocytes: 100 nM LY354740 alone and co-applied with 30 μM LY487379, 3 slices, n = 21; 300 nM LY354740 alone and co-applied with 30 μM LY487379, 5 slices, n = 56; 1 μM LY354740 alone and co-applied with 30 μM LY487379, 3 slices, n = 49; Neuron: 1 μM LY354740 alone and co-applied with 30 μM LY487379, 3 slices, n = 36). Compound application is indicated by the striped (LY354740) and grey (LY4872379) bars. **d** Bargraphs summarise results from a number of experiments demonstrating antagonism of the Group II agonist effect. The two bars on the left display ÄF% changes in calcium fluorescence upon application of 1 μM of the Group II agonist LY354740 alone and in conjunction with 1 μM of the Group II antagonist LY341495. The two bars on the right display ÄF% changes in calcium fluorescence upon application of 1 μM of the Group II agonist LY354740 alone and in conjunction with 5 μM MPEP and 100 μM suramin. **e** Upper traces display fluorescence over time for four example astrocytes from a slice from a wild-type (IP3R2+/+) mouse with responses to Group II agonist (1 μM) and glutamate (100 μM). Traces below show responses from astrocytes in a slice from an IP3R2(-/-) knock-out mouse. Bargraphs to the right summarise a number of experiments. Bars in **c** and **d** represent the mean % response (±SEM) of the fluorescence, **p < 0.01, ***p < 0.001.

Are mGlu2 receptors themselves able to directly activate astrocytes? To address this question, we monitored intracellular calcium levels in both VB neurons and astrocytes in an acute *in vitro* thalamic slice preparation. In the presence of TTX to block neuronal activity, a sub-maximal concentration of the Group II orthosteric agonist induced increases in intracellular calcium levels compared to baseline when applied alone (300 nM, ratio amplitude 0.016 ± 0.002, n = 56 astrocytes from 5 slices, Fig. 3a–c). Application of the mGlu2 PAM alone had no effect on intracellular calcium levels (data not shown). Upon co-application of the mGlu2 PAM with the agonist there was a significant potentiation in astrocytic intracellular calcium levels in the same astrocytes (300 nM LY354740 plus 30 μM LY487379, ratio amplitude 0.035 ± 0.003, n = 56 astrocytes from 5 slices, *p* < 0.001; Fig. 3a–c). This Group II mGlu receptor effect could be reversed upon co-application of 1 μM of the Group II antagonist LY341495 (1 μM LY354740, 2.11 ± 0.45 ΔF% change; 1 μM LY354740 plus 1 μM LY341495, 0.28 ± 0.17ΔF% change, n = 10 astrocytes from 5 slices, *p* < 0.01; Fig. 3d). Co-application of the Group II agonist with 5 μM 2-Methyl-6-(phenylethynyl)pyridine (MPEP) and 100 μM suramin had no effect (1 μM LY354740 alone, 3.14 ± 0.30 ΔF% change; 1 μM LY354740 plus 5 μM MPEP and 100 μM suramin, 2.96 ± 0.30 ΔF% change, n = 106 astrocytes from 4 slices; Fig. 3d), ruling out any mGlu5 or purine receptor involvement. Furthermore, there was no change in the intracellular calcium levels in neurons in the same slices when a maximal concentration of the Group II orthosteric agonist was applied alone (1 μM, ratio amplitude 0.004 ± 0.006, n = 36 neurons from 3 slices; Fig. 3a–c) nor when co-applied in the same slices with the mGlu2 PAM (1 μM LY354740 plus 30 μM LY487379, ratio change 0.005 ± 0.003, n = 36 neurons from 3 slices, *p* > 0.05; Fig. 3a–c). G_q_-protein coupled receptor-dependent calcium fluxes in astrocytes are a result of the inositol-1,4,5-triphosphate receptor (IP3R) activation resulting in the release of endoplasmic reticulum calcium ions into the cytosol (Sharp et al., 1999; Holtzclaw et al., 2002; Hertle and Yeckel, 2007; Petravicz et al., 2008). Astrocytes predominantly express the IP3R2 subtype (Petravicz et al., 2008). We therefore tested the effects of the mGlu2 agonist on acute slices from IP3R2 -/- knockout mice. A maximal concentration (1 μM) of the orthosteric agonist LY354740 was applied to Fluo-4 loaded slices from wild-type (IP3R2+/+) and knock-out (IP3R2-/-) mice. A maximal concentration of glutamate (100 μM) that also activates other calcium signalling pathways such as ionotropic receptors was subsequently applied to the same slice to provide an internal control. In slices from wild-type animals, both LY354740 and glutamate elicited robust astrocyte calcium elevations (LY354740: 5.12 ± 0.65%, Glutamate 7.41 ± 0.60, n = 188 astrocytes, 6 slices). However in slices from knock-out mice, while glutamate elicited calcium elevations (5.60 ± 0.35%), responses to LY354730 were abrogated (1.05 ± 0.18%, n = 100 astrocytes from 5 slices Fig. 3e). The initial Ca^2+^ peak induced by glutamate is abolished in the IP3R2 knock-out preparation, and can likely be attributed to Group I mGluR activation, whose signal transduction pathway is mediated via Gq. The remaining glutamate effect in the IP3R2 knock-out preparation can be attributed largely to activation of ionotropic glutamate receptors (Höft et al., 2014), as application of ionotropic glutamate receptor antagonists (NBQX and D-AP5) reduced the calcium associated fluorescence by 79% (data not shown); whilst the initial Ca2+ peak, abolished, and this can likely be attributed to Group I mGluR activation. Together, these data show that mGlu2 receptors elicit functional astrocyte responses via IP3R2 mediated calcium release; an effect traditionally associated with G_q/11_ coupled metabotropic receptors, as opposed to the G_i/o_ coupled mGlu2 receptor. However, the same metabotropic receptor, when expressed in different cell types/brain areas, is able to couple with alternate G-proteins: GABA_B_ receptors have been reported to couple to both G_i/o_ and G_q_ (Gould et al., 2014, Mariotti et al., 2016) and D1 receptors to G_s_, G_olf_ and G_q_ proteins (Lee et al., 2004). Furthermore, GABA_B_ receptor activation, usually assumed to be coupled via G_i/o_, induces calcium elevations in VB thalamus (Gould et al., 2014). As well as confirming an astrocytic locus for thalamic mGlu2 action, this represents a novel mechanism of astrocytic activation.

### 3.3 Astrocytes gate neuronal responses to somatosensory stimulation

**Fig. 4:**
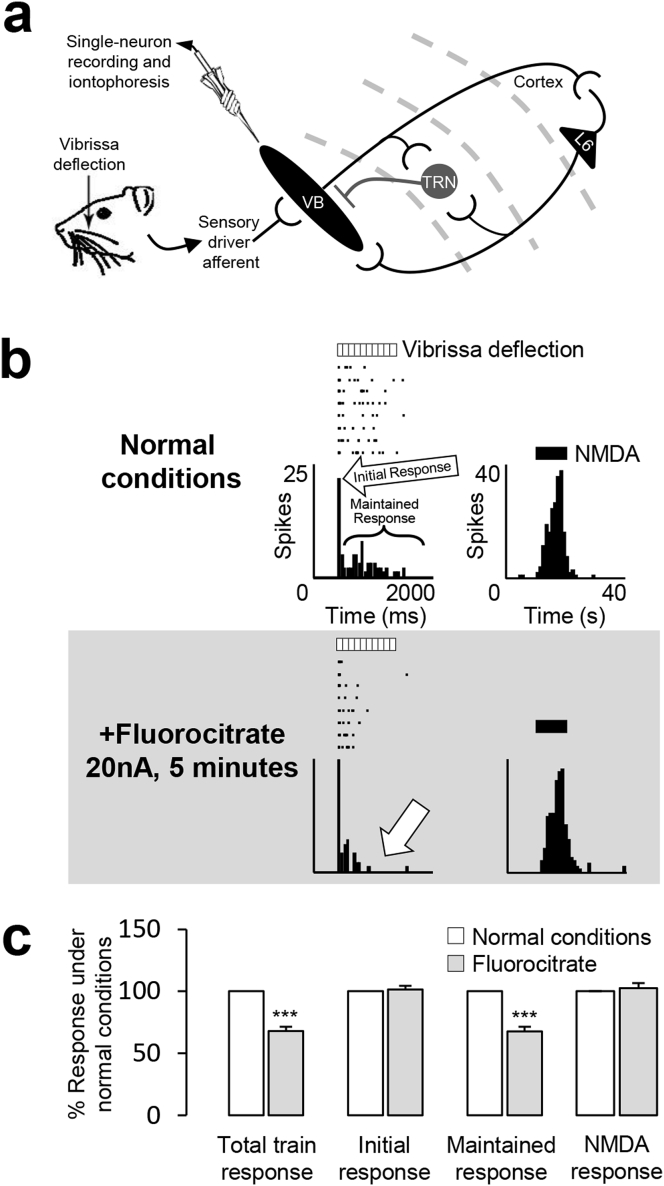
**Astrocyte inactivation attenuates the maintained component of VB neuron responses without affecting responses to NMDA. a** Circuitry between the TRN and VB with recording site indicated. **b** Raster displays and peristimulus time histograms (PSTHs) of responses of a VB neuron (CVB142c) to either train stimulation of a single vibrissa (50 ms bins, 8 trials) or iontophoretic application of NMDA (15 nA; 1s bins, 2 trials) under normal conditions and in the presence of fluorocitrate (20 nA; 5 min). **c** Bars represent the mean % response (±SEM) under normal conditions (100%) and in the presence of fluorocitrate to train stimulation (total, initial and maintained) of single vibrissae (n = 16) and NMDA (n = 11). ***p < 0.001.

Does this mechanism modulate thalamocortical responses to sensory stimulation in an *in vivo* system (Fig. 4a)? To test this question, we first assessed whether astrocytes contribute to the generation of VB neuron responses to physiological somatosensory stimulation. A recording electrode with iontophoretic capabilities was advanced into the VB of rats, and vibrissae were deflected as required to generate physiologically relevant activity. Observed waveforms were similar to those previously published (Salt, 1989), and were not perturbed under experimental conditions. Fluorocitrate selectively inhibits glia by interfering with the astrocytic tricarboxylic acid cycle (Fonnum et al., 1997), which is used to generate energy in the form of guanosine triphosphate (GTP). Upon local application of fluorocitrate, a reduction in neuronal responses to repetitive 10 Hz stimulation (1s duration) of the principal vibrissae was observed. Specifically, the maintained component of the neuronal response profile was significantly reduced (68 ± 4% of control, n = 16 from 9 rats, *p* < 0.001), whereas the initial component remained unaffected (101 ± 3% of control, n = 16 from 9 rats, *p* > 0.05) (Fig. 4b,c). The maintained component of neuronal responses to vibrissa stimulation comprises an NMDA-mediated contribution under normal physiological conditions (Salt, 1986). However, neuronal responses to exogenous NMDA application were unaffected in the presence of fluorocitrate (102 ± 4% of control, n = 11 from 5 rats, *p* > 0.05) (Fig. 4b right panels; Fig. 4c), thus indicating that inhibition of astrocyte function does not impact directly on NMDA receptor responses or upon post-synaptic neuronal excitability. Furthermore, as the initial component of neuronal responses to vibrissa stimulation was reliably present upon each stimulus presentation (see raster plot in Fig. 4b) the impact of fluorocitrate on neurotransmitter release can be considered minimal. The reduction of the maintained neuronal response component to vibrissae stimulation observed in the presence of fluorocitrate could therefore be attributable to the attenuation of an astrocytic mechanism of synaptic modulation independent of a direct effect on the postsynaptic VB neuron.

### 3.4 mGlu2 receptors modulate synaptic transmission at the TRN-VB synapse via an astrocyte-dependent mechanism

**Fig. 5:**
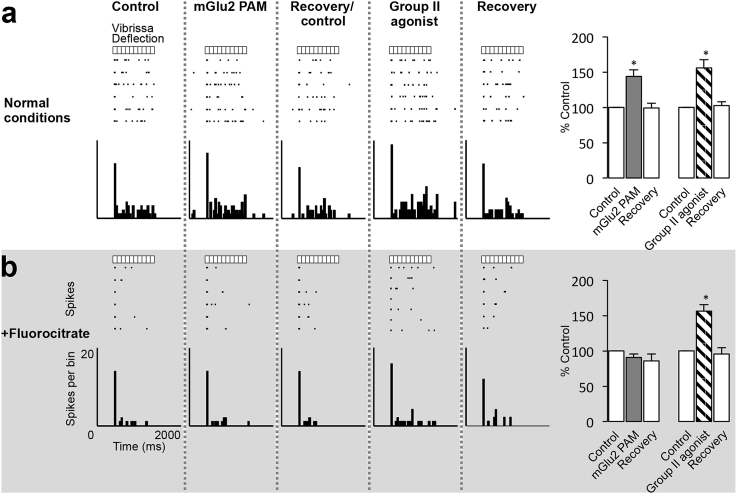
**Astrocyte inactivation attenuates the mGlu2 component of the Group II effect on sensory inhibition in the VB. a** Raster displays and PSTHs of responses of a VB neuron (CVB138a) to train stimulation (50 ms bins, 6 trials) of a single vibrissa under normal conditions and in the presence of fluorocitrate (20 nA; 10 min) during a control period, upon iontophoretic application of either LY487379 (50 nA, 2 min) or LY354740 (50 nA, 2 min), and during recovery. Abscissa indicated on the bottom left raster and PSTH plot applies to all plots. **b** Bars represent the mean % of control (±SEM) of responses to train stimulation of single vibrissae (n = 6) to application of either LY487379 or LY354740 under normal conditions and in the presence of fluorocitrate. *p < 0.05.

Consistent with previous findings (Copeland et al., 2012), local application of both the Group II orthosteric agonist LY354740 and the mGlu2 PAM LY487379 were able to significantly increase neuronal responses to 10 Hz train stimulation of principal vibrissae under normal conditions (LY354740: 156 ± 12% of control, n = 6 from 4 rats, *p* < 0.05; LY487379: 144 ± 10% of control, n = 6 from 6 rats, *p* < 0.05; Fig. 5a). However, in the same population of neurons in the presence of fluorocitrate, the effect of the mGlu2 PAM was completely abolished (91 ± 5% of fluorocitrate control, n = 6 from 4 rats, *p* > 0.05; Fig. 5b) whereas the Group II mGlu receptor orthosteric agonist effect remained (156 ± 9% of fluorocitrate control, n = 6 from 6 rats, *p* < 0.05; Fig. 5b). Fluorocitrate inhibits formation of the energy source GTP (Fonnum et al., 1997), which is required for mGlu2 receptor signal transduction (Niswender and Conn, 2010). From this selective attenuation of the mGlu2 PAM effect upon inhibition of astrocyte function we can infer that mGlu2 receptor modulation of the TRN-VB synapse function is astrocyte-dependent. Furthermore, we can attribute the remaining Group II mGlu receptor orthosteric agonist effect to activation of neuronal mGlu3 receptors localised on presynaptic TRN terminals (Tamaru et al., 2001, Turner and Salt, 2003). Thus we have now shown that mGlu2 receptor-mediated effects upon somatosensory transmission within the VB are astrocyte dependent under physiological conditions.

## 4 Discussion

**Fig. 6:**
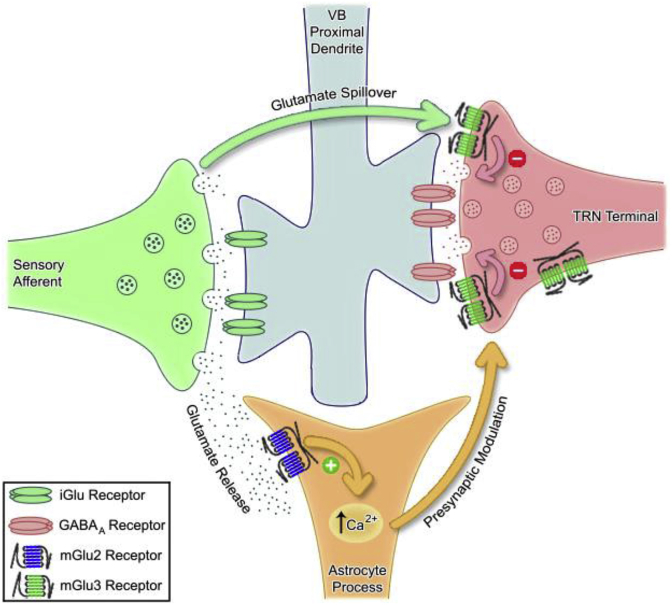
**Summary diagram of Group II mGlu receptor localizations in the VB, and their effects upon synaptic transmission.** Using selective pharmacological compounds, we have been able to show that mGlu2 receptors are likely located on astrocytic processes surrounding the TRN-VB synapse, whilst mGlu3 receptors are likely located on the TRN terminals themselves in the VB. Activation of astrocytic mGlu2 receptors likely facilitates elevations in intracellular calcium levels (indicated by a green plus), which may lead to presynaptic modulation of the TRN-VB synapse, whilst neuronal mGlu3 receptor activation is thought to decrease GABAergic transmission (indicated by the red minus signs). Both of the Group II mGlu receptor subtypes are likely activated via glutamate spillover from the synapse formed between the sensory afferent and the VB proximal dendrite upon physiological sensory stimulation (Copeland et al., 2012). (For interpretation of the references to colour in this figure legend, the reader is referred to the web version of this article.)

By using selective pharmacological tools in complementary *in vitro* and *in vivo* preparations we have been able to identify a novel mechanism of mGlu2 receptor-mediated astrocytic activation. In summary, our *in vitro* experiments showed that selective potentiation of mGlu2 receptor activity contributes to reducing inhibitory transmission at the TRN-VB synapse, and delineate the anatomical localisation of these mGlu2 receptors to astrocytes, whose processes are known to co-localise with TRN terminals on the soma and/or proximal dendrites of VB neurons (Ralston, 1983, Ohara and Lieberman, 1993) (Fig. 6). Our *in vivo* experiments extend our *in vitro* findings, showing that selective potentiation of mGlu2 receptor activity leads to an astrocyte-dependent increase in VB neuron responsiveness to somatosensory stimulation in a physiological context. This mechanism likely occurs upon physiological sensory stimulation as previously described, with the source of endogenous glutamate being glutamate spillover from the sensory afferent terminals activating mGlu2 receptors localised on the glial processes (Copeland et al., 2012). Therefore, we provide the first evidence that physiological activation of astrocytic mGlu2 receptors leads to concomitant modulation of thalamic processing of sensory inputs. Furthermore, previously the mGlu3 and mGlu5 receptor subtypes have been shown to be expressed in astrocytes (Niswender and Conn, 2010), with the mGlu5 subtype shown to mediate sensory driven activation of thalamic astrocytes (Parri et al., 2010). We have now shown functional astrocytic mGlu2 receptors are also able to elicit an increase in astrocytic intracellular calcium levels.

Whilst it has been previously demonstrated that astrocytes can act as a primary source of glutamate (Parri et al., 2001), we have now shown for the first time that astrocytes can themselves be activated via mGlu2 receptors. The functional outcome of this mechanism facilitates disinhibition of the postsynaptic VB neuron via action at the presynaptic TRN bouton, thus increasing the responsivity of the VB neuron to sensory stimulation, as opposed to providing a direct postsynaptic excitatory innervation (Parri et al., 2001). We are able to delineate this by drawing together results from different experimental paradigms. Firstly, neither the selective glial inhibitor fluorocitrate nor the Group II orthosteric agonist or mGlu2 PAM perturbed VB neuronal responses to exogenous NMDA (Copeland et al., 2012), indicating that neither normal astrocytic function nor Group II/mGlu2 receptor activation directly impinges upon the postsynaptic excitability of the VB neuron. The latter of these results also provides evidence against the involvement of somatodendritically expressed mGlu2 receptors (Watanabe and Nakanishi, 2003), which corresponds with ultrastructural evidence indicating a lack of Group II mGlu receptor expression by sensory thalamic neurons (Alexander and Godwin, 2006). Furthermore, Group II mGlu receptor activation reduces IPSPs evoked in VB neurons upon stimulation of the TRN without an effect on postsynaptic membrane properties in an *in vitro* thalamic slice preparation (Turner and Salt, 2003), indicative of a presynaptic mechanism of action. Taken together, these results indicate that astrocytic mGlu2 receptors act to modulate sensory-evoked inhibition in the VB via a mechanism independent of a direct effect on the postsynaptic neuron, and is therefore likely a presynaptic mechanism acting to reduce inhibitory synaptic transmission from the TRN to the VB. Indeed, astrocytes have been shown to release adenosine, which can lead to the opening of neuronal potassium channels (Winder et al., 1996) and subsequent modulation of neuronal excitability and action potential propagation. This mechanism of astrocyte-neuron signalling would thus reduce calcium influx at the TRN terminal and subsequent vesicle fusion with the presynaptic membrane. Such a non-neuronal dependent component of synaptic transmission could be occurring upon activation of astrocytic mGlu2 receptors (Fig. 6).

We are also able to infer that there is an mGlu3 receptor component to the overall Group II mGlu receptor activity on reducing inhibitory synaptic transmission at the TRN-VB synapse: when examining the *in vivo* electrophysiological data, whilst the selective glial inhibitor fluorocitrate was able to eliminate the mGlu2 PAM effect on sensory-evoked inhibition in the VB, there was a remaining Group II orthosteric agonist effect, indicative of an mGlu3 receptor component likely mediated by neuronal mechanisms. Indeed, there is anatomical evidence that mGlu3, but not mGlu2, receptors are located on TRN terminals within the VB (Tamaru et al., 2001), and that they are able to increase responses to sensory stimulation via a reduction in inhibition arising from the TRN (Turner and Salt, 2003). The function of these presynaptic mGlu3 receptors, which inhibit GABAergic transmission from the TRN by increasing K^+^ conductance (Alexander and Godwin, 2006, Cox and Sherman, 1999), is opposite to that of Group I mGlu receptors (mGlu1 & mGlu5), which depolarise TRN neurons by decreasing K^+^ conductance (Cox and Sherman, 1999) and activating a Ca^2+^-dependent non-selective cation conductance (Neyer et al., 2016). This duality in glutamatergic signalling would suggest a state-dependent reciprocal role of glutamate within the VB-TRN complex, which may act in concert to support complex behaviours. It is important to note that there is also evidence from ultrastructural studies that indicate both Group II mGlu receptors may be localised on glial processes surrounding the TRN-VB synapse (Ralston, 1983, Ohara and Lieberman, 1993, Liu et al., 1998, Mineff and Valtschanoff, 1999). However, due to a lack of commercially available mGlu3 selective ligands, we are unable to investigate whether there is an mGlu3 astrocytic component to the overall Group II mGlu receptor effect on sensory-evoked inhibition in the VB.

## 5 Conclusions

In conclusion, our findings, at the cellular and network levels, provide causal support for the hypothesis that mGlu2 receptor modulation of the TRN-VB synapse is astrocyte dependent. This is the first evidence that mGlu2 receptors are able to activate astrocytes, and represents a tripartite signalling pathway that modulates sensory processing in the thalamus. The TRN is responsible for ensuring synchronous activity across almost all functional modalities (Pinault, 2004) through inhibitory and disinhibitory circuits. Modulation of thalamic inhibitory processing via this novel astrocyte-dependent mechanism therefore represents an integral component of thalamic function thought to be of importance in the control of sensory discriminative processes (Copeland et al., 2012). This mechanism likely functions within thalamic circuitry to enable relevant information to be discerned from background activity, and would thus also be important in the understanding of synaptic processes underlying attention and cognition. This mechanism may therefore be an important potential therapeutic target in conditions where perturbed inhibitory systems have been hypothesised as contributory factors, such as in epilepsy and schizophrenia (Huguenard, 1999, Rub et al., 2003, Barbas and Zikopoulos, 2007, Pinault, 2011).

## Author contributions

CS Copeland conceived and designed experiments, collected, analysed and interpreted data, drafted the article, and approved the final version to be submitted. TM Wall collected, analysed and interpreted data, revised the article critically for important intellectual content, and approved the final version to be submitted.

Sims RE collected, analysed and interpreted data, revised the article critically for important intellectual content, and approved the final version to be submitted.

SA Neale conceived and designed experiments, revised the article critically for important intellectual content, and approved the final version to be submitted.

E Nisenbaum conceived and designed experiments, revised the article critically for important intellectual content, and approved the final version to be submitted.

HR Parri conceived and designed experiments, collected, analysed and interpreted data, revised the article critically for important intellectual content, and approved the final version to be submitted. TE Salt conceived and designed experiments, revised the article critically for important intellectual content, and approved the final version to be submitted.

## Conflict of interest

The authors declare no conflicts of interest.
